# DNA barcoding allows identification of undescribed crab megalopas from the open sea

**DOI:** 10.1038/s41598-021-99486-4

**Published:** 2021-10-18

**Authors:** Elena Marco-Herrero, Jose A. Cuesta, J. Ignacio González-Gordillo

**Affiliations:** 1 Instituto Universitario de Investigación Marina (INMAR), Universidad de Cádiz, Avda. República Saharaui, s/n, 11519 Puerto Real, Cádiz, Spain; 2 Centro oceanográfico de Cádiz, (IEO-CSIC), Puerto Pesquero, Muelle de Levante, s/n, 11006 Cádiz, Spain; 3 Instituto de Ciencias Marinas de Andalucía (ICMAN-CSIC), Avda. República Saharaui, 2 , 11519 Puerto Real, Cádiz, Spain

**Keywords:** Developmental biology, Zoology

## Abstract

Megalopas of 15 brachyuran crab species collected in the open sea plankton, and unknown until now, were identified using DNA barcodes (COI and 16S rRNA). Specimens belonging to the families Portunidae, Pseudorhombilidae and Xanthidae (Crustacea, Decapoda, Brachyura), and corresponding to the species *Achelous floridanus*, *Arenaeus mexicanus*, *Callinectes amnicola*, *C. arcuatus*, *C. ornatus, C. toxones*,* Charybdis (Charybdis) hellerii*, *Portunus hastatus*, *Thalamita admete*, *Scopolius nuttingi, Etisus odhneri*, *Liomera cinctimanus*, *Neoliomera cerasinus*, *Pseudoliomera variolosa*, and *Williamstimpsonia stimpsoni,* are described and illustrated, and compared with other congeneric species previously described. We also provide a new geographical record for *N. cerasinus* and the most remarkable features for each species.

## Introduction

One of the most relevant and influential scientific method in the last decade is DNA barcoding. It is considered an effective tool for species identification in different animal groups^1,2^ and is becoming increasingly common in biodiversity and conservation science^3,4^. Since its introduction 17 years ago, DNA barcoding has been widely applied by taxonomists as indicated by hundreds of published taxonomic studies^5–8^.

In this context, crustaceans, that represent one of the most diverse metazoan groups from a morphological and ecological point of view^9^ with more than 67,000 described species so far^10^, are an interesting target taxon for DNA barcoding because they are not always easy to identify by traditional approaches and usually require the help of highly trained taxonomists^11^. One of the biggest problems is to identify the larval stages of this group, because the larvae are distinguishable but not easily matched with the correct adult form^12,13^. Therefore, this problem causes obstacles in studies such as plankton ecology or population connectivity^14,15^.

This is the case of crabs. Most brachyuran crabs pass through a planktonic larval period with two phases, zoea and megalopa, which are remarkably different from each other and from the adult form^16,17^. The megalopa is a planktonic phase characterized by the existence of functional pleonal swimming appendages, the pleopods, while the anterior thoracic appendages (the maxillipeds) assume functions as mouthparts^18,19^. This stage usually looks for structurally complex habitats, which can provide refuge and food^20^ and many studies refer to the megalopa as settle and recruitment phase^21,22^.

Particularly, the identification of megalopas from plankton samples based on morphological characters is a difficult task and in many cases is not possible do it at genus or species level^19,23^. In this sense, DNA barcoding provide rapid and accurate identifications of plankton specimens^24–27^, being the only limitation the need of previous knowledge of DNA markers for the species in accessible databases as Genbank or BOLD.

In this study DNA barcoding was used to identify the megalopa stage of brachyuran crabs from open sea plankton across the world, collected in the context of the MALASPINA and MAF research projects. In this work, we focus on describing unknown megalopas belonging to the families Portunidae Rafinesque, 1815, Pseudorhombilidae Alcock, 1900 and Xanthidae MacLeay, 1838. Portunidae are among the most diverse and species rich groups of brachyuran crabs with a worldwide distribution, including many taxa which are of high ecological and economical significance^28^. Portunids larvae identification is particularly difficult^29,30^ because the larvae of different species are so similar, that it is difficult to tell species apart other than by examination of minute characteristics^31,32^. On the other hand, representatives of the Xanthidae present a circumtropical distribution while Pseudorhombilidae are known almost exclusively from waters of the Americas. Commonly known as mud, pebble, rubble, or blackfingered crabs^33^, are familiar forms in many marine settings although many species remain poorly described and lack detailed illustrations^34^.

Once identified by DNA barcoding, in the present work a morphological description and illustrations are carried out for the megalopas of 15 species, namely the portunids: *Achelous floridanus* (Rathbun, 1930*)*, *Arenaeus mexicanus* (Gerstaecker, 1856), *Callinectes amnicola* (de Rochebrune, 1883)*, Callinectes arcuatus* Ordway, 1863, *Callinectes ornatus* Ordway, 1863, *Callinectes toxones* Ordway, 1863, *Charybdis* (*Charybdis*) *hellerii* (A. Milne-Edwards, 1867), *Portunus hastatus* (Linnaeus, 1767), and *Thalamita admete* (Herbst, 1803), the xanthids: *Etisus odhneri* Takeda, 1971, *Liomera cinctimanus* (White, 1847), *Neoliomera cerasinus* Ng, 2002, *Pseudoliomera variolosa* (Borradaile, 1902), and *Williamstimpsonia stimpsoni* (A. Milne-Edwards, 1879), and the pseudorhombilid: *Scopolius nuttingi* (Rathbun, 1898).

## Results

A total of 462 megalopas were collected in the course of two different projects, 375 in the MALASPINA Expedition, and 87 in the MAF cruise.

These megalopas were initially sorted according to their general external morphology in main morphotypes groups and, from each of these; representatives were selected for DNA barcoding. Partial mitochondrial COI and/or 16S rRNA gene sequences were obtained for 139 larvae, leading to the identification of 67 megalopas from 34 species.

### DNA barcode identification

Among a total of 139 megalopas analysed by DNA barcoding, 72 could not be identified to species level only based on morphological features, since their DNA barcodes did not allow for accurate identification, and therefore cannot be described. The other 67 were identified as belonging to 34 different species of the families Calappidae De Haan, 1833 [in De Haan, 1833–1850] (4), Cryptochiridae Paulson, 1875 (3), Dromiidae De Haan, 1833 [in De Haan, 1833–1850] (1), Eriphiidae MacLeay, 1838 (1), Grapsidae MacLeay, 1838 (6), Homolidae De Haan, 1839 [in De Haan, 1833–1850] (1), Ocypodidae Rafinesque, 1815 (1), Panopeidae Ortmann, 1893 (1), Parthenopidae MacLeay, 1838 (2), Portunidae (9), Pseudorhombilidae (1), and Xanthidae (5).

Of these 34 species, only 3*, Menippe nodifrons* Stimpson, 1859, *Eurypanopeus abbreviatus* (Stimpson, 1860), and *Homola barbata* (Fabricius, 1793), have its megalopa previously described^35–37^, and when they were compared no significant differences were found, for this reason do not need to be redescribed. Their sequences have been deposited in Genbank: *M. nodifrons* (16S: MW264136, COI: MW264437), *E. abbreviatus* (16S: MW264137, COI: MW264438), and *H*. *barbata* (COI: MW264436). The present work focuses on the 31 megalopas of Portunidae, Pseudorhombilidae and Xanthidae assigned to 15 species (Table 1).

**Table 1:** Identification of the megalopas collected in the in the MALASPINA 2010–2011 and MAF 2015 cruises based on 16S and COI barcodes.

Megalopa ID	Species identification	16S (%, mt, bp)	References	COI (%, mt, bp)	References	Megalopa sequences	
						16S	COI
MF10, MF20-23	*Achelous floridanus*	DQ388058 (99, 2, 519)	Mantelatto et al.^90^*^1^	ACC4724 (99, ?, ?)	BOLD (Unpublished)	MW264138	MW264439
ML30	*Arenaeus mexicanus*	JX123471 (100, 0, 553)	Zupolini et al (unpublished)	JX123449 (99, 3, 665)	Zupolini et al. (unpublished)	MW264139	MW264440
MF24-27	*Callinectes amnicola*	No Seq		MG462523 (99, 7, 658)	Windsor et al.^91^	MW264140	MW264441
ML09	*Callinectes arcuatus*	KY940141 (100, 0, 545)	Lopes et al. (unpublished)	MG462537 (100, 0, 658)	Windsor et al.^91^	MW264141	MW264442
ML20	*Callinectes arcuatus*	KY940141 (99, 1, 545)	Lopes et al. (unpublished)	MG462539 (99, 5, 658)	Windsor et al.^91^	MW264142	MW264443
ML28	*Callinectes arcuatus*	KY940141 (100, 0, 545)	Lopes et al. (unpublished)	MG462539 (99, 7, 658)	Windsor et al.^91^	MW264143	MW264444
ML73	*Callinectes arcuatus*	KY940141 (100, 0, 545)	Lopes et al. (unpublished)	MG462539 (99, 4, 589)	Windsor et al.^91^	MW264144	MW264445
ML74	*Callinectes arcuatus*	KY940141 (100, 0, 545)	Lopes et al. (unpublished)	MG462537 (100, 0, 611)	Windsor et al.^91^	MW264145	MW264446
ML71	*Callinectes ornatus*	KY940120 (100, 0, 406)	Lopes et al. (unpublished)	–		MW264146	–
ML103	*Callinectes toxotes*	DQ407681 (100, 0, 547)	Robles et al.^92^	MG462541	Windsor et al.^91^	MW264147	MW264447
ML10, ML16	*Charybdis (Charybdis) hellerii*	KX060470 (100, 0, 535)	Negri et al.^93^	KX060349 (100, 0, 665)	Negri et al.^93^	MW264148	MW264448
ML36, ML95, ML96	*Portunus (Portunus) hastatus*	FM208780 (99, 1, 555)	Schubart and Reuschel^94^	No Seq		MW264149	MW264449
ML59	*Thalamita admete*	FJ152163 (100, 0, 518)	Mantelatto et al.^38^	KT365766 (99, 5, 657)	Evans ^39^*^2^	MW264150	MW264450
ML14	*Scolopius nuttingi*	MF490190 (99, 1, 480)	Mantelatto et al.^95^*^3^	MF490096 (100, 0, 653)	Mantelatto et al.^95^*^3^	MW264151	MW264451
ML49	*Etisus odhneri*	HM798455 (99, 3, 432)	Lai et al.^40^	–		MW264152	–
ML52	*Liomera cinctimanus*	HM798486 (99, 2, 462)	Lai et al.^40^	–		MW264153	–
ML43	*Neoliomera cerasinus*	HM798518 (99, 3, 417)	Lai et al.^40^	–		MW264154	–
ML65	*Pseudoliomera variolosa*	HM978551 (100, 0, 433)	Lai et al.^40^	–		MW264155	–
ML29	*Williamstimpsonia stimpsoni*	KF682971 (100, 0, 454)	Thoma et al.^34^	KF682971 (100, 0, 524)	Thoma et al.^34^	MW264156	MW264452

Indicating: (1) species identified, (2) accession codes of the Genbank and BOLD sequences (when there is more than one sequence it was selected the longest), including in brackets: % of similarity with megalopa sequence, divergence (as number of mutations) and number of base pairs compared, and (3) accession codes of the megalopas sequences in Genbank. ML and MF refer megalopa codes from MALASPINA 2010–2011and MAF 2015 expeditions, respectively; *mt* mutations, – sequence no obtained, *No Seq* no sequence fit > 90% in databases, *^1^, as *Portunus floridanus*; *^2^, as *Thalamita gatavakensis*; *^3^, as *Micropanope nuttingi.*

While 16S sequences were obtained for all 31 megalopas analyzed, COI could not be reared for five species, specially xanthoids (see Table 1). Using 16S sequences eight species were identified fitting 100% with sequences in Genbank, but only four with COI in Genbank and BOLD, because it is a marker with more intraspecific variability.

The results of the BLAST search show some inaccuracies. In the case of the sequences of the megalopa of a *Thalamita* Latreille, 1829 species we obtained two sequences fitting 100% in 518 bp and 531 bp for the 16S sequence. The first one corresponded to *Thalamita admete*, specimen ULLZ 4382 from South Africa, obtained by Mantelatto et al.^38^, the second one to *Thalamita gatavakensis* Nobili, 1906, specimen UF: 17,469 collected in Lizard Island (Australia), obtained by Evans^39^. In the case of COI sequence, only one sequence fitted 99% (5 mutations in 657 bp) and belong to UF: 17,486, another specimen of *Thalamita gatavakensis* from Lizard Island, also obtained by Evans^39^. Taken into account that the megalopa was collected close to South Africa we have considered *T. admete* as the right identification, but future studies are needed to clarify the relationship between *T. admete* and *T. gatavakensis*. Similarly, for one megalopa its 16S sequence fit 99% (only 2 mutations in 462 bp) with one sequence of *Liomera cinctimanus* (as *Liomera cinctimana*) obtained by Lai et al.^40^. However, other two sequences are deposited in Genbank as belonging to *L. cinctimanus*, with 19 mutations in 518 bp and 21 mutations in 519 bp, both obtained by Wetzer et al.^41^. In all cases, the specimens were collected in Guam, but they clearly do not belong to the same species according to the differences found, higher than intraspecific variability. The megalopa was collected close to South Africa, therefore so far from Guam, but within of the wide distribution of this species. *Scopolius nuttingi*, represent a third similar case, since two sequences of 16S were found fitting 99% (only 1 mutation) with that of the megalopa. The two sequences are identical, but one is identified as *Micropanope nuttingi* (MF490190 obtained by Mantelatto et al.^8^), and another one as *M. scultipes* (KT279707 obtained by Faria et al.^42^). However, a third sequence (GU144437), shorter and with only one mutation in the same position, is identified as *M. nuttingi* by Felder and Thoma^43^ and the COI sequence of the megalopa fit 100% with two sequences of *M. nuttingi.* For these reasons we have considered *Scopolius nuttingi* as the correct identification.

### New record

**Figure 1:**
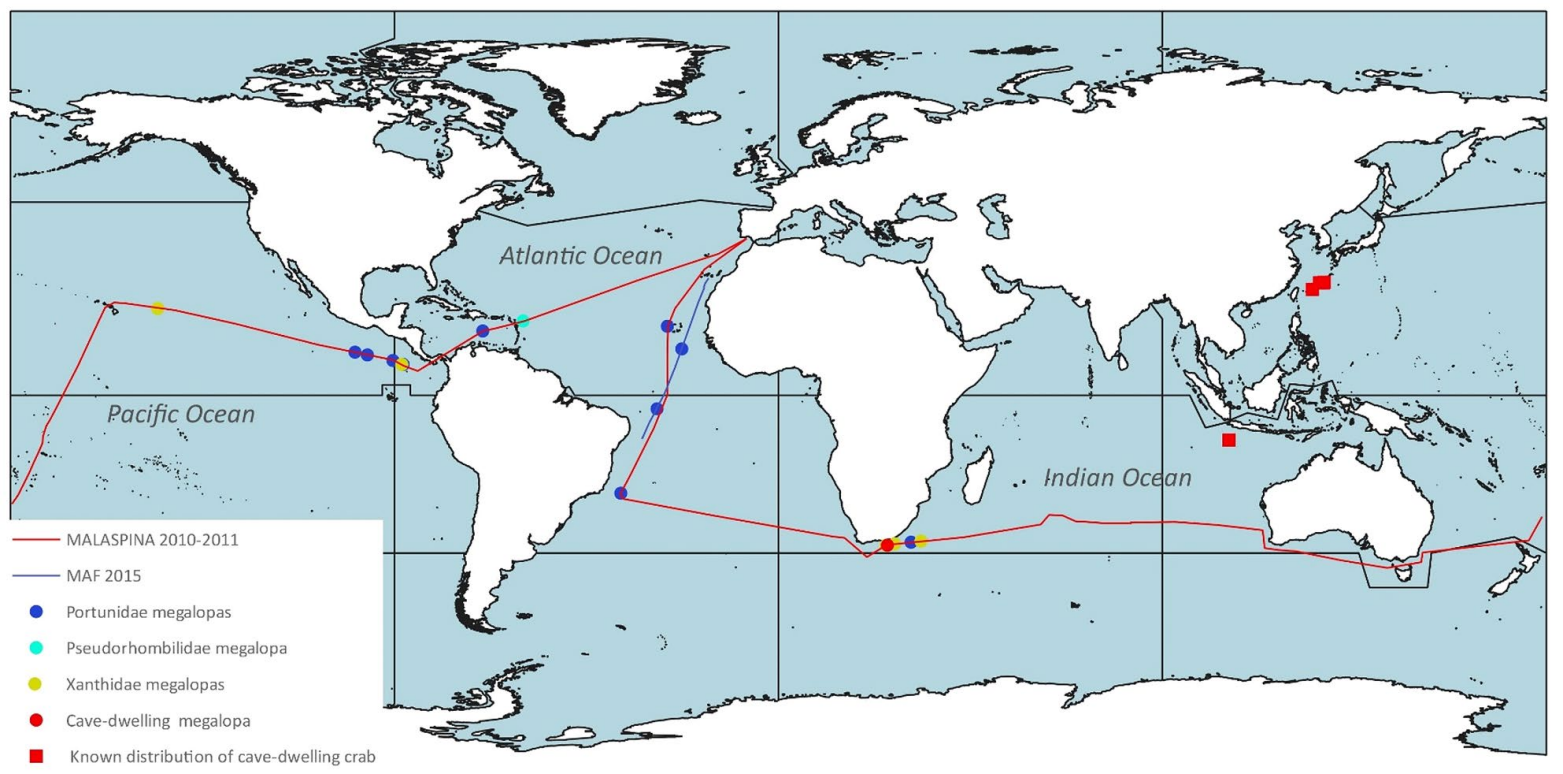
Map of megalopa samples taken and cruise tracks during the MALASPINA 2010–2011 and MAF 2015 expeditions, and the known distribution of the cave-dwelling crab, *Neoliomera cerasinus*.

A single cave-dwelling megalopa of *Neoliomera cerasinus* was caught on 13 February 2011 in South African coast (35° 08′ 10′′ S, 25° 33′ 47′′ E). This specimen constitutes the first occurrence of *Neoliomera cerasinus* from the Indian Ocean coast of South Africa. Previous records of this cave-dwelling xanthid crab from the Indian Ocean were from Christmas Island: Thunderdome Cave [topotypical locality] and West White Cave^44^, and in the Pacific Ocean in Kumejima Island, Ryukyu Islands, Japan^45^ and Okinawa Island and Shimoji Island, Ryukyu Islands^46^, expanding widely the distribution of this species to the opposite extreme of the Indian Ocean (see Fig. 1).

### Megalopas descriptions

Family Portunidae Rafinesque, 1815

Genus *Achelous* De Haan, 1833

**Figure 2:**
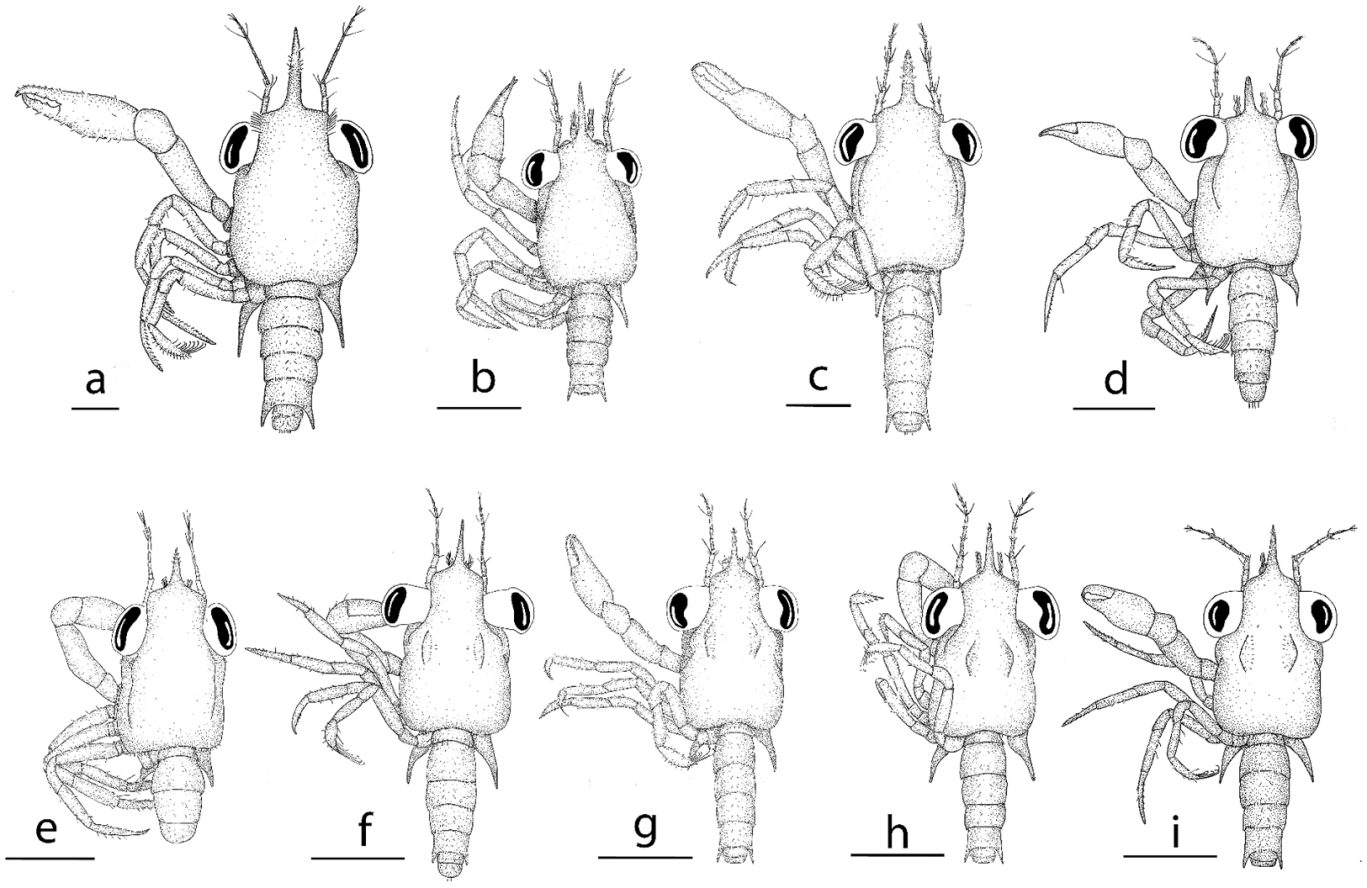
Dorsal view of megalopa: (**a**) *Achelous floridanus*; (**b**) *Arenaeus mexicanus*; (**c**) *Portunus hastatus*; (**d**) *Charybdis (Charybdis) hellerii*; (**e**) *Thalamita admete*; (**f**) *Callinectes toxotes*; (**g**) *Callinectes arcuatus;* (**h**) *Callinectes ornatus;* (**i**) *Callinectes amnicola*. Scale bars: 1 mm.

**Figure 3:**
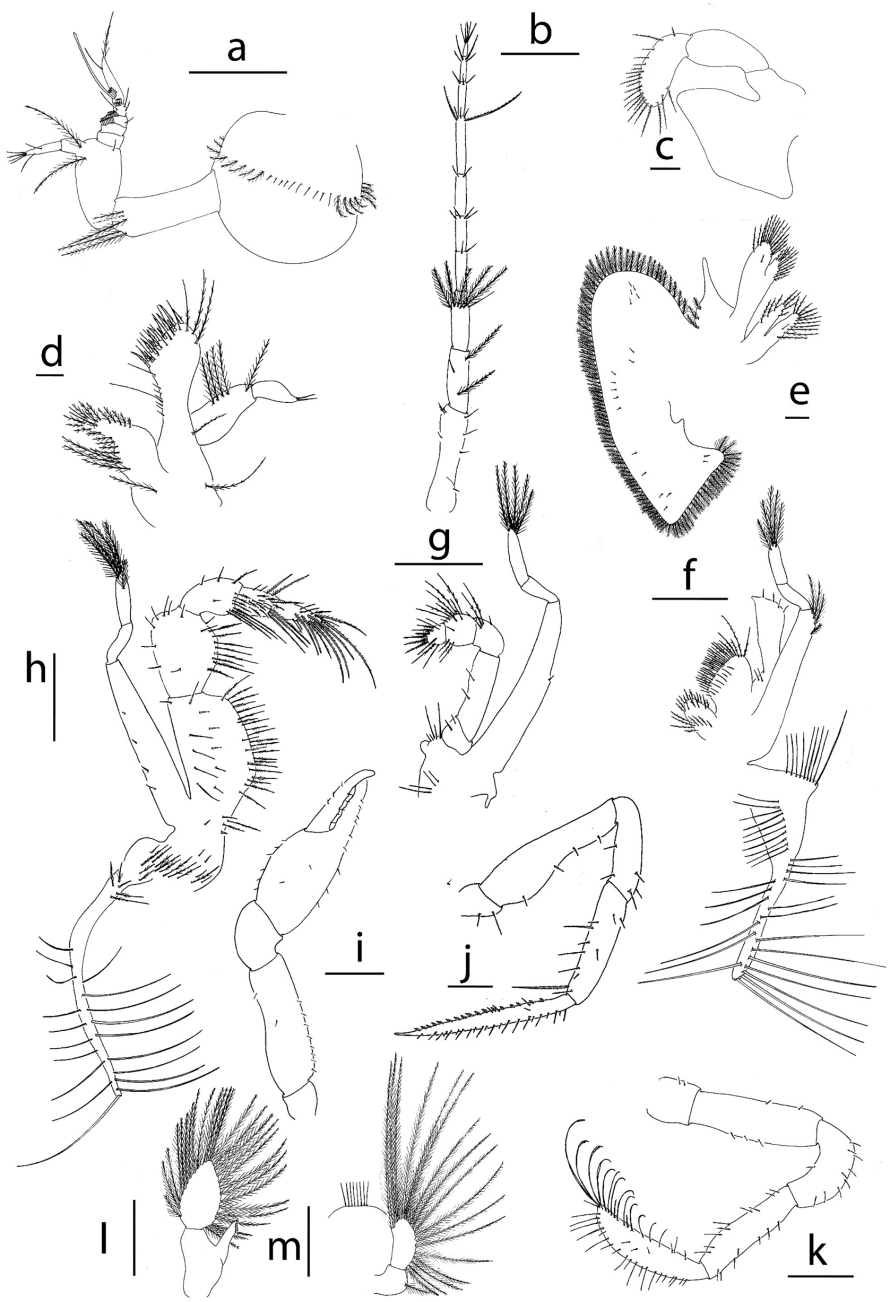
Appendages of *Achelous floridanus* megalopa: (**a**) antennule; (**b**) antenna; (**c**) mandible; (**d**) maxillule; (**e**) maxilla; (**f**) first maxilliped; (**g**) second maxilliped; (**h**) third maxilliped; (**i**) cheliped (**j**) second pereiopod; (**k**) fifth pereiopod; (**l**) second pleopod; (**m**) uropod. Scale bars: (**c**–**e**), 0.1 mm; (**a**, **b**, **h**, **j**–**m**) 500 µm; (**i**) 1 mm.

**Figure 4:**
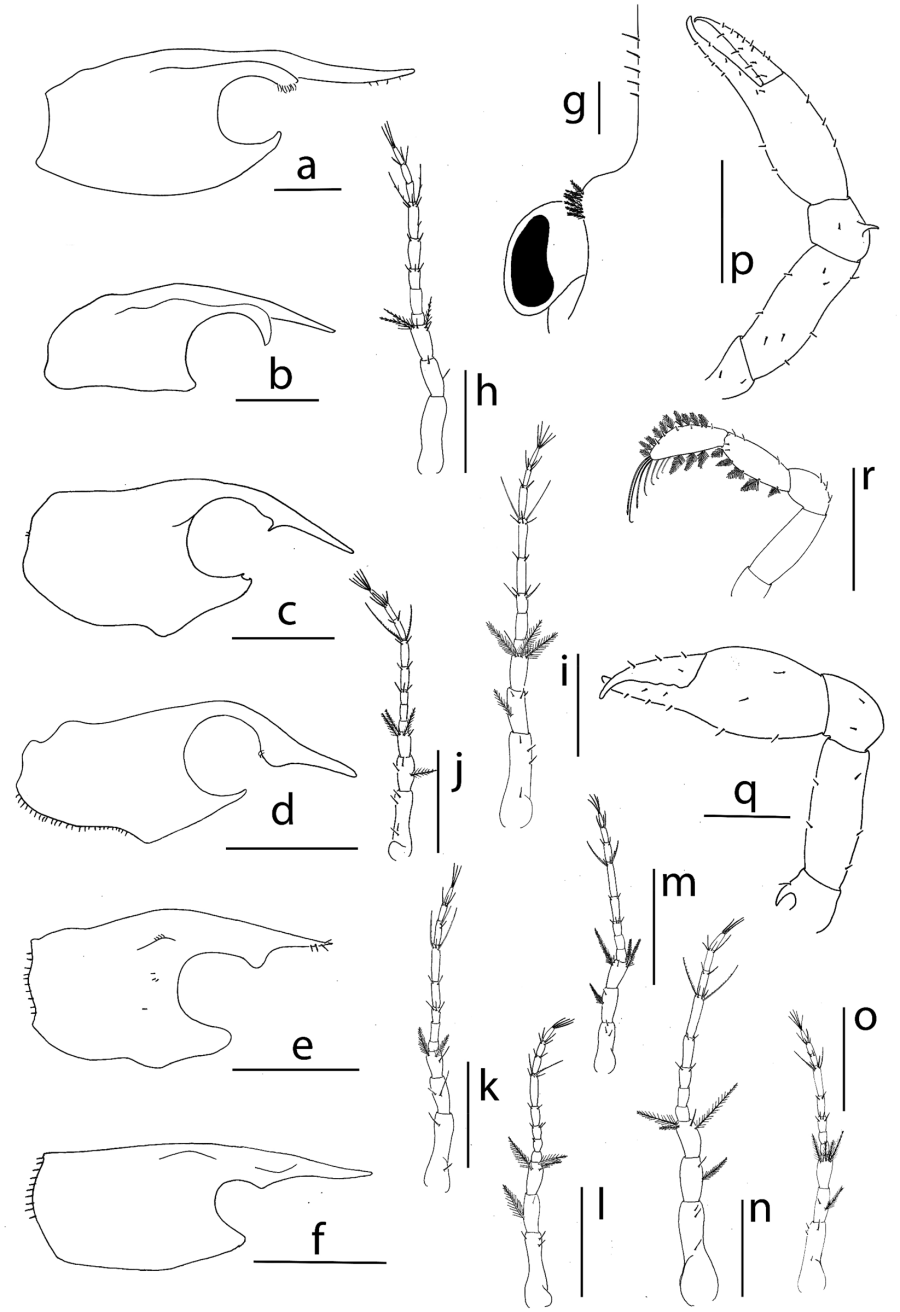
Lateral view of megalopa: (**a**) *Achelous floridanus*; (**b**) *Arenaeus mexicanus*; (**c**) *Portunus hastatus*; (**d**) *Charybdis (Charybdis) hellerii*; (**e**) *Thalamita admete*; (**f**) *Callinectes toxotes*. Rostrum dorsal view: (**g**) *Achelous floridanus*. Antenna: (**h**) *Arenaeus mexicanus*; (**i**) *Portunus hastatus*; (**j**) *Charybdis (Charybdis) hellerii*; (**k**) *Thalamita admete;* (**l**) *Callinectes toxotes*; (**m**) *Callinectes arcuatus*; (**n**) *Callinectes ornatus;* (**o**) *Callinectes amnicola*. Cheliped: (**p**) *Portunus hastatus*; (**q**) *Callinectes amnicola*. Fifth pereiopod: (**r**) *Callinectes amnicola*. Scale bars: (**a**–**f**, **p**–**r**, **I**) 1 mm; (**h**–**e**, **m**–**n**, **o**) 0.5 mm.

*Achelous floridanus* (Rathbun, 1930)

(Figs. 2a, 3a–m, 4a, g, 8a)

Size: CL: 3.86 ± 0.15 mm; CW: 2.84 ± 0.16 mm; n = 5.

*Cephalothorax* (Figs. 2a, 4a, g) Longer than broad, with long, thin and slightly curved upwards spine rostral with ventrally minute setae; orbital region with 7 plumose setae; a pair of lobes on the mesobranchial regions with hepatic regions moderately inflated; setation as drawn; dorsal organ present; eyes stalked.

*Antennule* (Fig. 3a) Peduncle 3-segmented with 16 plumose + 10 simple setae around the first segment, 4 plumose + 1 simple setae on second segment and 2 long plumose + 1 simple setae on the distal segment; primary flagellum with 5 annuli, with 0, 0, 1, 3, 1 simple + 1 sparsely setae and 0, 18, 16, 12, 6 aesthetascs respectively; accessory flagellum without annuli with 1 medial + 1 subterminal + 4 terminal simple setae.

*Antenna* (Fig. 3b) Peduncle 3-segmented with 5 simple + 1 plumodenticulate, 2 plumose + 1 simple, 1 simple + 5 plumose setae; flagellum 8-segmented with 0, 2, 4, 2, 3 + 2 (long serrated), 3, 4, 4–5 simple setae respectively.

*Mandible* (Fig. 3c) Palp 3-segmented with 22–24 plumodenticulate marginal setae on distal segment.

*Maxillule* (Fig. 3d) Coxal endite with 22–23 setae; basial endite with 6 small setae + 2 long sparsely plumose lateral setae and 22–24 cuspidate setae plus other one on the dorsolateral margin; endopod 2-segmented with 4 + 1 long plumose setae on proximal segment and 2 terminal simple setae on distal segment; long exopodal sparsely plumose seta present.

*Maxilla* (Fig. 3e) Coxal endite bilobed with 12 + 7 terminal plumose setae; basial endite bilobed with 10–12 + 15–16 sparsely plumodenticulate setae; endopod unsegmented with 3 short plumodenticulate setae on dorsal margin; exopod (scaphognathite) with 105–108 marginal plumose setae plus 19–20 setae on lateral surface.

*First maxilliped* (Fig. 3f) Epipod triangular shaped with 9 proximal plumodenticulate and 29–30 distal long simple setae; coxal endite with 20–24 plumose setae; basial endite with 37–42 sparsely plumodenticulate setae; endopod unsegmented with 2 + 4 simple setae; exopod 3-segmented with 4 plumose distal setae on proximal segment and 5 terminal plumose setae on distal segment.

*Second maxilliped* (Fig. 3g) Epipod reduced without setae; coxa with 2 + 2 + 4 simple setae; endopod 5-segmented with 2 simple, 2 simple, 4 long simple, 12 plumodenticulate and 11 (6 cuspidate and 5 plumodenticulate) setae, respectively; exopod 3-segmented with 1 medial simple seta on proximal segment and 5 terminal plumose setae on distal segment.

*Third maxilliped* (Fig. 3h) Epipod with 5 plumodenticulate + 20–21 long simple setae; protopod with 16–20 plumodenticulate setae; endopod 5-segmented with 41–45, 22–27, 17–19, 18–20, 13–15 sparsely plumose setae respectively; exopod 3-segmented with 4 marginal simple setae on proximal segment and 6 terminal plumose setae on distal segment.

*Pereiopod* (Figs. 3i–k, 8a) Cheliped setation as drawn; pereiopod II with hook coxal and pereiopod III with small tubercle on coxal segment; pereiopods II-IV with propodial setae present; pereiopods II-V thin and setose, inner margin of dactyl with 12–13 stout ventral spines; dactylus of pereiopod V with 3 long setae (feelers) plus 5 shorts + 3 long small setae like feelers.

*Sternum* (Fig. 8a) Maxilliped sternites completely fused with 10 simple setae plus one central pair of setae, cheliped sternites with 7 simple setae each, pereiopod sternites 2–3 with small tubercle with 7 simple setae each; pereiopod sternite IV with a long pointed posterolateral spine with 4 setae; sternal sutures are interrupted medially.

*Pleon* (Fig. 2a) Six pleonites; pleonite I without setae; setation of pleonites II-VI as shown; pleonite VI reduced.

*Pleopods* (Fig. 3l, m) Present on pleonites II-VI; endopods unsegmented with 4–5 cincinuli; exopod unsegmented with 36–37, 36–38, 32–34, 27–29 long plumose natatory setae; uropod 2-segmented, proximal segment with 1 seta, distal segment with 17–18 terminal plumose natatory setae.

*Telson* (Fig. 3m) Reduced, subquadrate, with 1 pair of dorsal setae and 7–8 setae on posterior margin.

Distinctive morphological features for *Arenaeus mexicanus, Portunus hastatus, Charybdis (Charybdis) hellerii*, *Thalamita admete, Callinectes amnicola*, *Callinectes arcuatus, Callinectes ornatus*, and *Callinectes toxotes,* are listed in the Table 2 and illustrated in the Figs. 2b–I, 4b–o, 8b–i.

**Table 2:** Meristic and morphological characters of the megalopa stage of 8 species of the family Portunidae: *Arenaeus mexicanus, Portunus (Portunus) hastatus, Charybdys (Charybdys) hellerii, Thalamita admete, Callinectes toxotes, Callinectes arcuatus, Callinectes ornatus,* and *Callinectes amnicola*.

	*A. mexicanus*	*P. hastatus*	*C. helleri*	*T. admete*	*C. toxotes*	*C. arcuatus*	*C. ornatus*	*C. amnicola*
CL (mm)	1.53	2.52 ± 0,04	1.89 ± 0.007	1.91	1,77	1.605 ± 0.05	1.67	1.67 ± 0.05
CW (mm)	1.06	1.72 ± 0,07	1.39 ± 0.02	1.26	1.26	1.09 ± 0.03	1.12	1.14 ± 0.02
**Antennule**								
Peduncle (s)	5,5,3	10,6,3	3,4,3	6,4,5	8,4,3	7,5,3	8,5,2	5,4,2
Primary flagellum (a)	0,10,10,10,3	0,16,14,10,6	0,12,12,8,6	0,14,14,5,5	0,10,10,8,4	0,8,8,8,4	0,10,8,8,5	0,10,8,8,4
Primary flagellum (s)	0,0,1,1,2	0,0,1,2,2	0,0,1,3,2	0,0,0,1,2	0,0,1,1,2	0,0,1,2,2	0,0,1,3,2	0,0,1,3,2
Accesory flagellum (s)	1 + 5	1 + 1 + 4	1 + 1 + 3	1 + 1 + 3	1 + 3	1 + 1 + 4	1 + 3	1 + 1 + 4
**Antenna**								
Peduncle (s)	0,2,5	3,3,4	4,3,4	2,2,3	3,2,4	3,2,4	3,1,4	4,2,5
Flagellum (s)	0,0,4,2,6,0,3,3	0,0,4,2,5,2,4,5	0,0,3,2,4,2,4,5	0,0,3,2,3,2,3,3	0,0–1,3,2,4,2,4,4	0,0,4,2,5,2,4,4	0,0,2,2,5,2,4,3	0,0,4,2,4,2,4,5
**Mandible**								
Palp (s)	0,0,23	0,0,16	0,0,14	0,13	0,0,11	0,0,13	0,0,13	0,0,11
**Maxillule**								
Coxal endite (s)	21	16–20	19	18	20	15–17	13	15
Basial endite (s)	27	30	26	27	29	28	26	22
Epipodal/exopodal (s)	+/−	+/+	−/+	+	+ /+	+	+	+/+
Endopod (s)	4,2	(1) 3,2	(1) 3,2	(1) 3,2	(1) 3,2	(1) 3,2	(1) 3,2	(1) 3,2
**Maxilla**								
Coxal endite (s)	14 + 7	6–10 + 6–10	8 + 6	7 + 7	6 + 5	8 + 6–7	7 + 7	8 + 6
Basial endite (s)	8 + 10	10 + 14	8 + 11	9 + 10	7 + 11	8–9 + 10–12	8 + 11	8 + 11
Endopod (s)	0	2–3	3	1	2	2	0	0
Scaphognathite (s)	88	90–91	66	63	77	66–72	73	69
Scaphognathite inner (s)	6	15	9	7	12	12–13	8	13
**First maxilliped**								
Epipod (s)	5 + 17	6 + 18–22	4 + 11	4 + 15	5 + 12	6 + 15	2 + 12	6 + 16
Coxal endite (s)	21	16–20	10	14	17	15	19	20
Basial endite (s)	43	40–43	27	26	26	30	23	30
Endopod (s)	8 + 1	2 + 4	3	3	6	5	4	1 + 7
Exopod (s)	1,4	4,5	1,5	1,4	1,5	1,5	0,4	1,5
**Second maxilliped**								
Epipod (s)	1	0	0	0	0	0	0	0
Coxa (s)	2	5	3	3	4	1	1	4
Endopod (s)	3,3,3,9,11	3,4–5,3,8–10,10–12	2,2,2,8,10	2,3,2,8,11	2,5,2,9,10	2,5,3,9,10	2,6,3,9,10	2,4,3,9,10
Exopod (s)	2,5	2,5	1,5	1,5	1,4	1,4	1,4	1,5
**Third maxilliped**								
Epipod (s)	3 + 16	7 + 16	6 + 8	5 + 8	7 + 16	5 + 15	10 + 10	10 + 20
Coxa and basis (s)	4	15	10	6	18	8–10	5	12
Endopod (s)	41,14,15,8,9	42,16–18,13,14–19–9	21,12,8,10,9	30,15,12,13,8	26,16,12,16,10	28,18,14,17,11	27,14,11,8	27,14,10,18,10
Exopod (s)	0,5	3,6	3,6	0,6	0,5	4,5	3,4	2,5
**Cheliped**								
Spine	Carpal	Carpal	–	–	–	–	–	–
Hook	Ischial	–	Ischial	–	Ischial	Ischial	Ischial	Ischial
**Pereiopods**								
Spine	2nd P coxal	2nd P coxal	2nd P coxal	–	–	–	–	–
Tubercles	–	3rd P coxal	3rd P coxal	–	–	–	–	–
**5th pereiopod**								
Feelers dactylus	3 + 6	3 + 6	3 + 2	No data	3 + 6	3 + 6	3 + 7	3 + 5
**Sternum**								
Setae	20	26	28	21	18	30	19	22
Tubercles	2nd–3rd sternite	3rd sternite	–	–	–	–	–	–
**Pleon**								
Posterior margin telson (s)	–	3	4	3	2	–	–	–
Pleopods (s)	28,28,27,25	29,26,25,21	23,25,25,21	18,21,20,19	21,18,18,22	21–23,20–23,19–20,19	19,18,18,18	21,21,20,20
Pleopods cincinuli	4–5	4–5	3–4	3–4	3–4	3–4	3–4	4–4–4–4
Uropod (s)	1,12	1,13–14	0–1,12–13	1,11	1,12	1,11	1,12	1,12

*a* aesthetacs, *s* setation, *P* pereiopod, + present, – absent.

Superfamily Xanthoidea MacLeay, 1838

Family Xanthidae MacLeay, 1838

Genus *Neoliomera* Odhner, 1925

**Figure 5:**
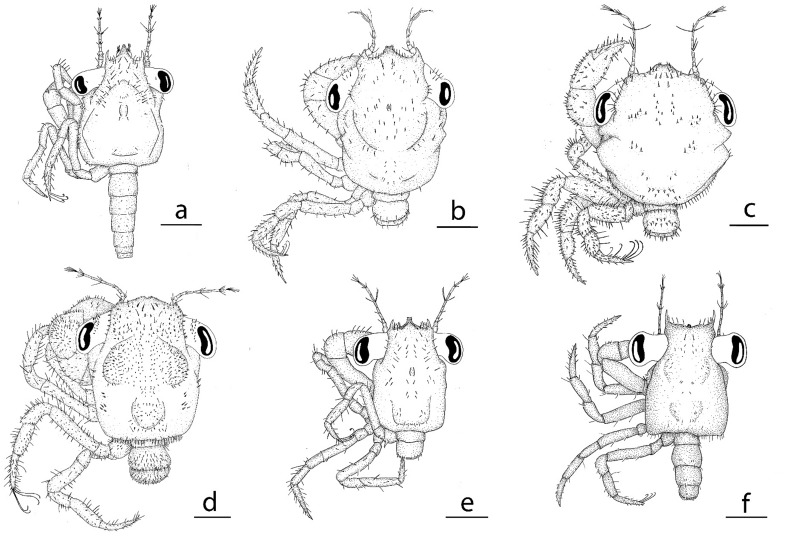
Dorsal view of megalopa: (**a**) *Neoliomera cerasinus*; (**b**) *Liomera cinctimanus*; (**c**) *Pseudoliomera variolosa*; (**d**) *Williamstimpsonia stimpsoni*; (**e**) *Scopolius nuttingi*; (**f**) *Etisus odhneri*. Scale bars: 0.5 mm.

**Figure 6:**
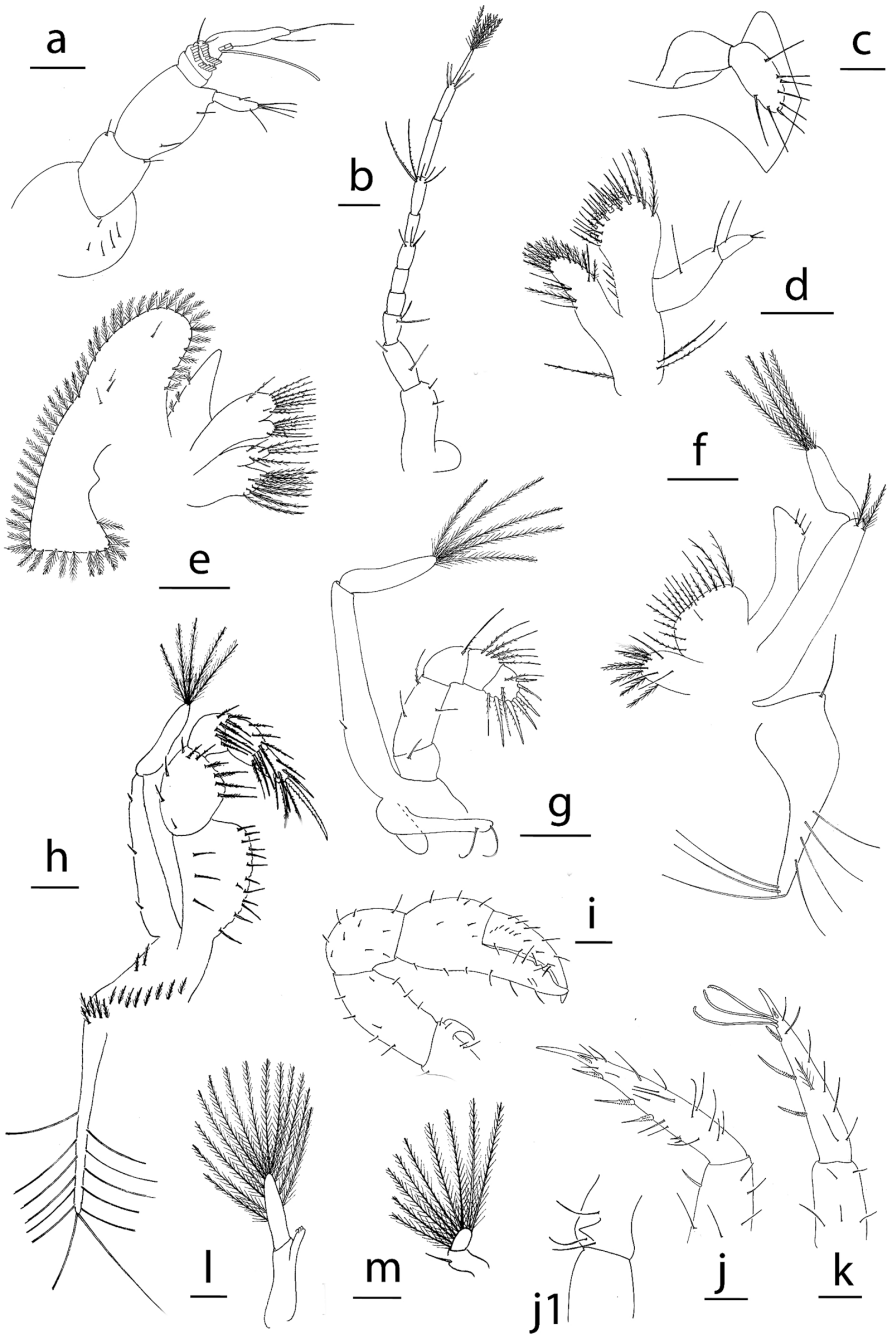
Appendages of *Neoliomera cerasinus*: (**a**) antennule; (**b**) antenna; (**c**) mandible; (**d**) maxillule; (**e**) maxilla; (**f**) first maxilliped; (**g**) second maxilliped; (**h**) third maxilliped; (**i**) cheliped; (**j**) dactylus of second pereiopod; (**j,1**) detail of dactylus of second pereiopod; (**k**), dactylus of fifth pereiopod; (**l**) second pleopod; (**m**) uropod. Scale bars: 0.1 mm.

**Figure 7:**
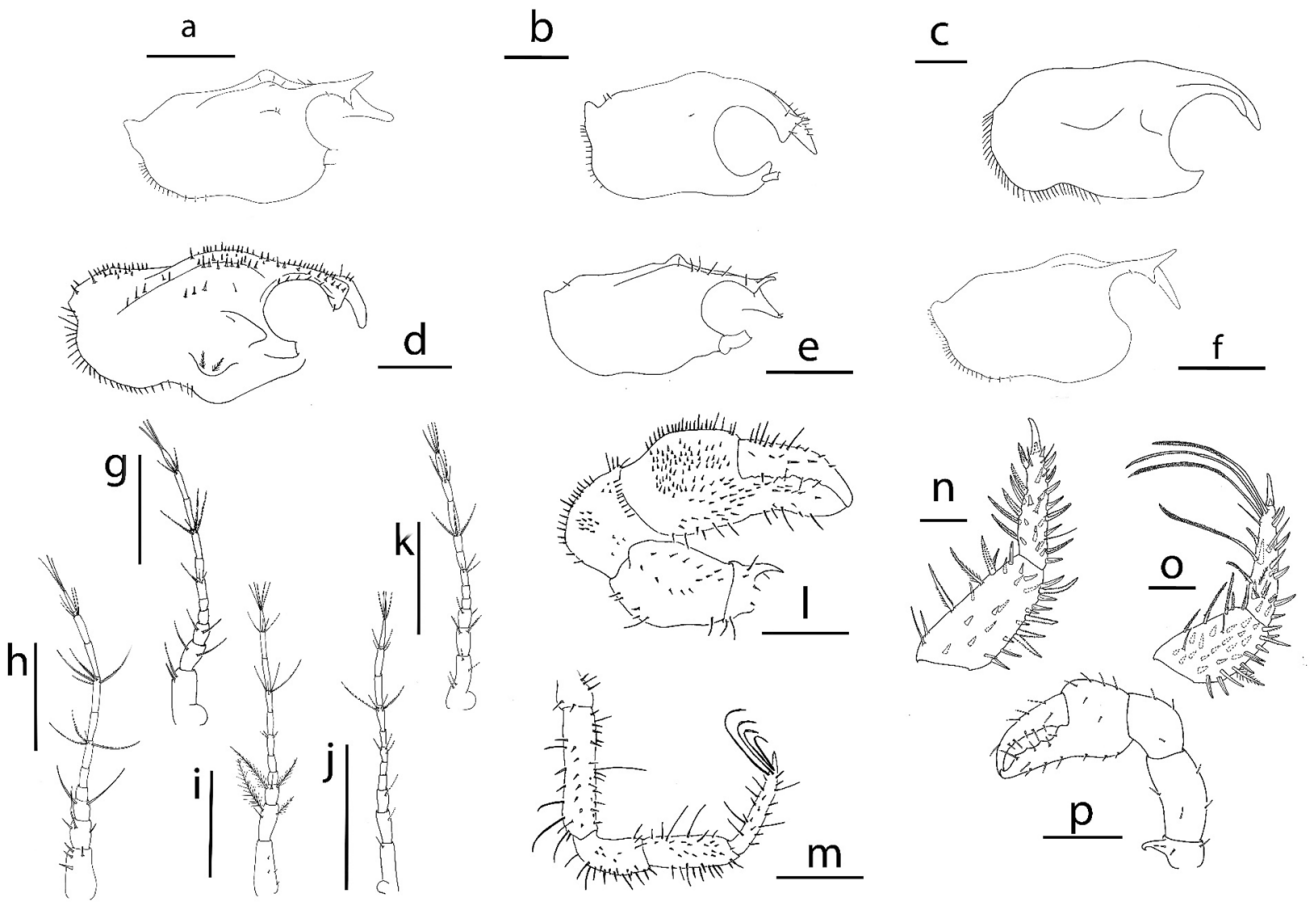
Lateral view of megalopa: (**a**) *Neoliomera cerasinus;* (**b**) *Liomera cinctimanus;* (**c**)* Pseudoliomera variolosa*; (**d**) *Williamstimpsonia stimpsoni*; (**e**) *Scopolius nuttingi;* (**f**)* Etisus odhneri.* Antenna: (**g**) *Liomera cinctimana;* (**h**) *Pseudoliomera variolosa*; (**i**) *Williamstimpsonian stimpsoni*; (**j**) *Scopolius nuttingi*; (**k**) *Etisus odhneri*. Cheliped: (**l**) *Williamstimpsonian stimpsoni*; (**p**) *Etisus odhneri.* Fifth pereiopod: (**m**) *Williamstimpsonian stimpsoni*; (**o**) *Pseudoliomera variolosa*. Dactylus of second pereiopod: (**n**) *Pseudoliomera variolosa*. Scale bars: 0.5 mm.

**Figure 8:**
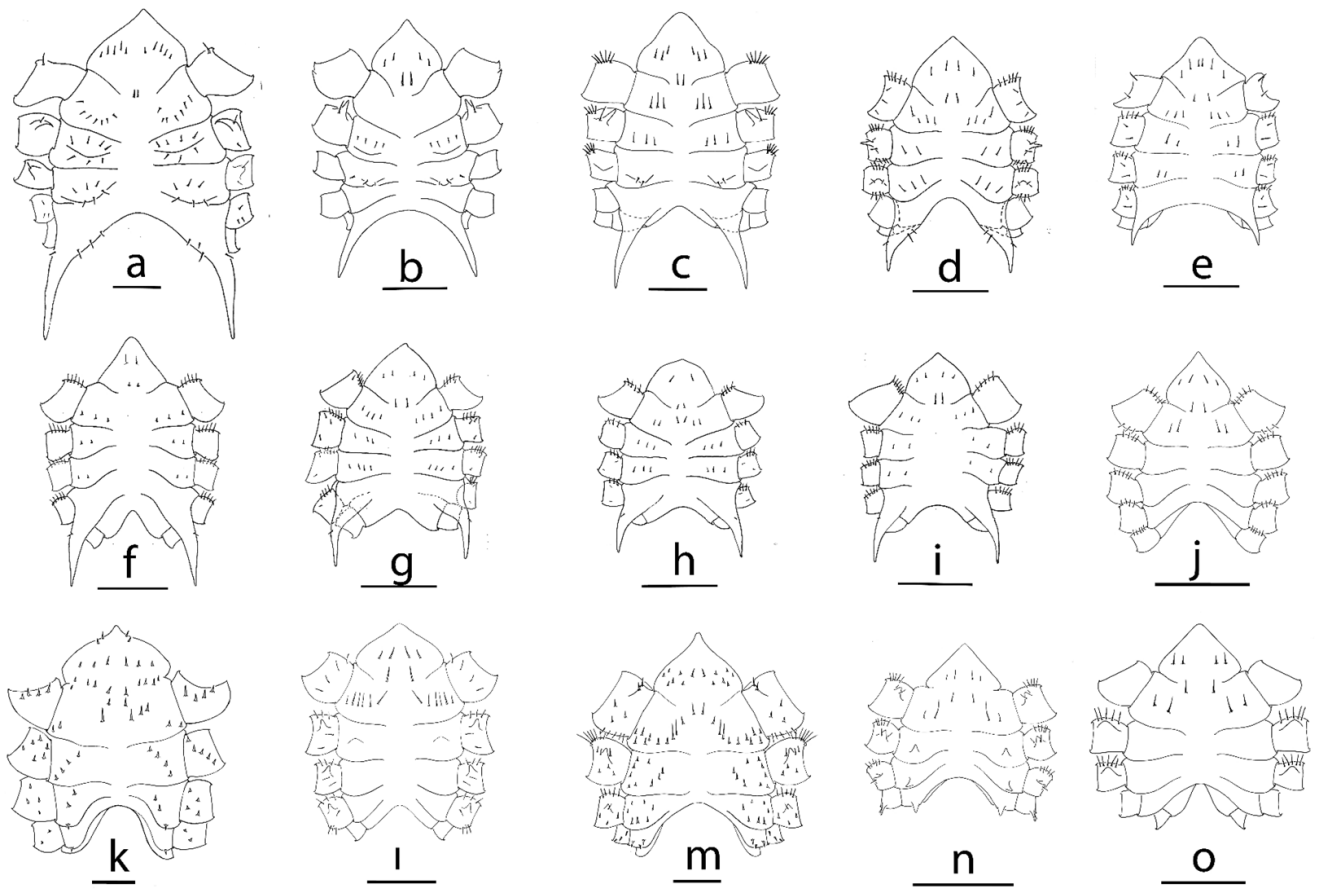
Sternum of megalopa: (**a**) *Achelous floridanus*; (**b**) *Arenaeus mexicanus*; (**c**) *Portunus hastatus*; (**d**) *Charybdis (Charybdis) hellerii*; (**e**) *Thalamita admete*; (**f**) *Callinectes toxotes*; (**g**) *Callinectes arcuatus;* (**h**) *Callinectes ornatus;* (**i**) *Callinectes amnicola*; (**j**) *Neoliomera cerasinus;* (**k**) *Liomera cinctimana;* (**l**) *Pseudoliomera variolosa*; (**m**) *Williamstimpsonian stimpsoni*; (**n**) *Scopolius nuttingi*; (**o**) *Etisus odhneri*. Scale bars: 0.5 mm.

*Neoliomera cerasinus* Ng, 2002

(Figs. 5a, 6a–m, 7a, 8j)

Size: CL: 1.34 mm; CW: 1.05 mm; n = 1.

*Cephalothorax* (Figs. 5a, 7a) Longer than broad; frontal margin with a pair of frontal submedian horns with a rostrum directed obliquely downwards; prominent tubercle on mesogastric, and a pair of lobes on protogastric and mesobranchial region; small tubercle on cardiac region; hepatic region inflated; dorsal organ present; eyes stalked.

*Antennule* (Fig. 6a) Peduncle 3-segmented with 5, 2, 2 simple setae respectively; primary flagellum with 4 annuli, with 0, 0, 2, 1 + 1 simple setae and with 0, 9, 7, 4 aesthetascs respectively; accessory flagellum without annuli, with 1 medial + 1 subterminal + 3 terminal simple setae.

*Antenna* (Fig. 6b) Peduncle 3-segmented with 2, 2, 2 simple setae; proximal segment with stout process; flagellum 8-segmented with 0, 0, 3 simple, 0, 2 long plumodenticulate + 3 short simple, 0, 4 simple, 4 simple setae respectively.

*Mandible* (Fig. 6c) Palp 2-segmented with 8 plumodenticulate terminal setae on distal segment.

*Maxillule* (Fig. 6d) Epipodal sparsely plumodenticulate seta present; coxal endite with 17 setae; basial endite with 4 small ventrolateral setae + 10 cuspidate and 9 plumodenticulate terminal setae + 3 long plumose setae; endopod 2-segmented with 2 simple setae on proximal segment and 1 long simple seta + 2 short terminal simple setae on distal segment; 2 long exopodal setae.

*Maxilla* (Fig. 6e) Coxal endite bilobed with 10 + 6 terminal plumose setae; basial endite bilobed with 6 + 10 sparsely plumodenticulate setae; endopod unsegmented with 3 short plumodenticulate setae on dorsal margin; exopod (scaphognathite) with 50–51 marginal plumose setae plus 2 + 2 setae on lateral surface.

*First maxilliped* (Fig. 6f) Epipod triangular shaped with 1 proximal and 7 distal long simple setae; coxal endite with 6 long plumose + 5 long simple setae; basial endite with 17 sparsely plumodenticulate setae; endopod unsegmented with 3 simple setae; exopod 2-segmented with 2 distal plumose setae on proximal segment and 4 terminal plumose setae on distal segment.

*Second maxilliped* (Fig. 6g) Epipod reduced with 2 setae; coxa without setae; endopod 5-segmented with 1 simple, 3 simple + 1 long plumodenticulate, 5 plumodenticulate and 9 (7 cuspidate and 2 plumodenticulate) setae, respectively; exopod 2-segmented with 1 medial simple seta on proximal segment and 5 terminal plumose setae on distal segment.

*Third maxilliped* (Fig. 6h) Epipod with 5 proximal plumose and 13 terminal long simple setae; protopod with 10 plumodenticulate setae; endopod 5-segmented, ventral margin of the proximal segment denticulate, and 16, 10, 7–8, 7, 7 setae respectively; exopod 2-segmented with 3 distal simple setae on proximal segment and 5 terminal plumose setae on distal segment.

*Pereiopods* (Fig. 6i–k, j1) Cheliped sparsely setose as shown, with prominent ischial curved hook; pereiopods II–V thin and setose, inner margin of dactyl with 3 stout spines and 2 shorter lateral spines; pereiopod II with a tubercle on ischial segment; dactylus of pereiopod V with 3 long setae (feelers).

*Sternum* (Fig. 8j) Maxilliped sternites completely fused with 4 simple setae, cheliped sternites with 2 simple setae each, pereiopod sternites 2–5 without setae; sternal sutures are interrupted medially.

*Pleon* (Fig. 5a) Six pleonites; setation of pleonites II-VI as shown; pleonite VI reduced.

*Pleopods* (Fig. 6l, m) Present on pleonites II-VI; endopods unsegmented with 3 cincinuli; exopod unsegmented with 16, 14, 14, 14 long plumose natatory setae; uropod 2-segmented, proximal segment with 1 seta, distal segment with 9 terminal plumose natatory setae.

*Telson* (Fig. 5a) Reduced, with 1 pair of dorsal setae.

Distinctive morphological features for *Liomera cinctimanus*, *Pseudoliomera variolosa*, *Williamstimpsonia stimpsoni*, *Scopolius nuttingi* and *Etisus odhneri* are listed in the Table 3 and illustrated in the Figs. 5b–f, 7b–p, 8k–o.

**Table 3:** Meristic and morphological characters of the megalopa stage of 4 species of Xanthidae: *Liomera cinctimanus, Pseudoliomera variolosa, Williamstimpsoni stimpsoni, Etisus odhneri,* and 1 species of Pseudorhombilidae: *Scopolius nuttingi*.

	*L. cinctimanus*	*P. variolosa*	*W. stimpsoni*	*E. odhneri*	*S. nuttingi*
CL (mm)	1.66	1.90	2.01	1.41	1.17
CW (mm)	.1.33	1.84	1.57	1.04	0.87
**Antennule**					
Peduncle (s)/sp	2 + 2sp,1 + 2sp,2	7sp,3sp,2sp	6,1,2	0,0,2 + 3lg	0,2,2 + 3lg + 3lg
Primary flagellum (a)	0,12,8,4	0,10,7,4	0,16,10,4	0,5,5,4	0,5,5,4
Primary flagellum (s)	0,0,1,2	0,0,1,2	0,0,2,2	0,0,2,2	0,0,1,2
Accesory flagellum (s)	1 + 1 + 3	1 + 1 + 2	1 + 1 + 3	1 + 1 + 3	1 + 1 + 4
**Antenna**					
Peduncle (s)/sp	2sp,2sp,2sp	4sp,3sp,2sp	4sp,2pl + 1sp,2pl + 1sp	4,2,2	1,1,1
Flagellum (s)	0,0,3,0,4,0,4,3	0,0,4,0,4,0,4,3	0,0,3,0,4,0,4,5	0,0,3,0,3,0,4,4	0,0,3,0,4,0,4,4
**Mandible**					
Palp (s)	0,11	0,12	0,0,14	0,9	0,10
**Maxillule**					
Coxal endite (s)	13	16	13	15	14
Basial endite (s)	23	27	21	21	15
Epipodal /exopodal (s)	+/+ (2)	+/+ (2)	+/+ (2)	+/+ (2)	+/+ (2)
Endopod (s)	2,3	(1) 2,3	(1) 2,3	2,3	2,3
**Maxilla**					
Coxal endite (s)	8 + 5	11 + 5	10 + 8	8 + 4	10 + 6
Basial endite (s)	5 + 10	7 + 11	6 + 10	6 + 9	6 + 9
Endopod (s)	2	8	1 + 6	5	1 + 4
Scaphognathite (s)	53	64	75	58	43
Scaphognathite, inner (s.)	2 + 3	3 + 5	3 + 4	1 + 3	2 + 2
**First maxilliped**					
Epipod (s)	1 + 6	1 + 11	1 + 12	1 + 5	1 + 8
Coxal endite (s)	5	16	11	10	9
Basial endite (s)	20	23	24	20	16
Endopod (s)	3	4	4	3	3
Exopod (s)	2,4	3,5	3,4	2,5	2,5
**Second maxilliped**					
Epipod (s)	0	12	9	2	2
Coxal endite (s)	1	1	0	1	0
Endopod (s)	2,3,1,4,8	2,3,1,5,8	2,5,1,6,9	2,2,1,6,8	3,3,1,5,8
Exopod (s)	2,4	2,4	2,5	2,4	2,4
**Third maxilliped**					
Epipod (s)	13 + 15	18 + 29	16 + 16	4 + 1 (broken)	4 + 13
Coxa and basis (s)	6	11	8	10	13
Endopod (s)	20,13,5,9,7	23,17,11,12,7	21,13,13,12,7	14,14,6,9,6	15,13,9,11,7
Exopod (s)	3,5	5,5	2,4	1,4	2,6
**Cheliped**					
Protuberance/tubercle	–	–	Coxal	–	Coxal
Hook	–	–	Ischial	Ischial	Ischial
**2nd, 3rd, 4th pereiopods**					
Spine	2nd–4th coxal-ischial	–	2nd–3rd coxal	2nd–3rd coxal	2nd–4th coxal
** 5th pereiopod**					
Feelers dactylus	3	1 + 4	4	3	3
**Sternum**					
Setae	18	48 sp	74 sp	10	10
Tubercles	2nd	–	–	–	–
Spine	–	–	–	–	2nd–4th
**Pleon**					
Posterior margin telson (s)	3	2 sp + 3	5lg	4	3
Pleopods (s)	19,21,16,16	25,24,21,17	23,23,22,19	18,18,17,15	17,16,15,14
Pleopods cincinuli	3–4	3–4	3	3	3–4
Uropod (s)	1,11	0,11	1,12–13	0,10	1,8–9

*a* aesthetacs, *lg* long, *pl* plumose, *s* setation, *sp* spine, + present, – absent.

### Remarks

Family Portunidae Rafinesque, 1815

The megalopas of Portunidae can be distinguished from those of other brachyurans for the following diagnostic combination of characters: presence of rostral spine projecting almost horizontally, the dactyl of the 5^th^ pereiopods paddle-like, pair of spines projecting posteriorly on 4^th^ sternite, and 5^th^ segment of pleon with lateral spines. Therefore, such features can also be observed in the megalopas of the following genera analyzed in this study: *Achelous* De Haan, 1833, *Arenaeus* Dana, 1851, *Callinectes* Stimpson, 1860; *Charybdis* De Haan, 1833; *Portunus* Weber, 1795 and *Thalamita* Latreille, 1829. Nevertheless, as different authors argue^29,30,47,48^ the identification of the megalopa stage of portunids at specific level is a difficult task, because to the close similarity of its morphologies that makes all larvae remarkably similar. This task is more complicated when the larvae are from planktonic samples. Next, we highlight the most distinctive morphological features for each studied species.

*Achelous floridanus* (Figs. 2a, 3a–m, 4a, g, 8a)

Mantelatto et al.^38^ proposed, and recently corroborated by Mantelatto et al.^8^, the resurrection of genus *Achelous* De Haan 1833, a reassignment of nine American species and eleven (of twelve) eastern Pacific species respectively, formerly treated as *Portunus* Weber, 1795*. Achelous* now contains a total of 21 American species, between them: *Achelous spinicarpus* Stimpson, 1871 and *Achelous spinimanus* (Latreille, 1819), the only ones whose megalopa have been described (Bookhout and Costlow^31^, as *Portunus spinicarpus;* and Negreiros-Fransozo et al.^49^, as *Portunus spinimanus,* respectively).

The megalopa morphology of *A. spinicarpus* and *A. spinimanus* is similar but both are different to *A. floridanus*. *A. spinicarpus* and *A. spinimanus* have 2 carpal spines on cheliped while they are absent in *A. floridanus*; the pair of spines projecting posteriorly on 4^th^ sternite on the sternum is much longer in *A. floridanus,* reaching the 3^rd^ pleonite but it is smaller in both remaining species; *A. floridanus* shows a characteristic rostrum curved upwards with numerous minute setae (Fig. 2a), and the orbital region presents 7 plumose setae (Fig. 4g), both characters are not present in *A. spinicarpus* and *A. spinimanus.* Finally, other important character that usually remains constant within the genus, such as the number of antennal segments and setation of endopod of the maxillule, is different between these species: *A. floridanus* shows an antenna 8-segmented while 7-segmented in *A. spinicarpus* and *A. spinimanus*.

*Arenaeus mexicanus* (Figs. 2b, 4b, h, 8b)

The genus *Arenaeus* encompasses only two species: *A. cribrarius* (Lamark, 1818) and *A. mexicanus*. Stuck and Truesdale^50^ describe the larval development of *A*. *cribanarius* and the diagnosis characters of the genus are summarized in an antennal flagellum 8-segmented, exopod of uropods with 1,12–13 setae, carpal spine and ischial hook on cheliped, coxal spine on 2^nd^ pereiopod and coxal tubercle on 3^rd^ pereiopod, and tubercles on 2^nd^–3^rd^ sternites.

The megalopa of *A. mexicanus* can be easy separated from that of *A. cribrarius* by the setation of antennal flagellum: 0,0,4,2,1 + 5,0,3,3 for *A. mexicanus* and 0,0,4,2,4,2,4,4 for *A. cribrarius*.

*Portunus hastatus* (Figs. 2c, 4c, p, 8c)

The genus *Portunus* includes about 100 species^51^ but the species with known megalopas only are *P. trituberculatus* (Miers, 1876), *P. pelagicus* (Linnaeus, 1758), and *P. gibbesii* (Stimpson, 1859) described by Kurata^29^, Yatsuzuka and Sakai^52^ and Negreiros-Fransozo et al.^49^, respectively.

Similar to the *Achelous* genus, the species included in *Portunus* show a high variability in certain morphological characters that have been considered key to characterize the genus. Therefore, there is not a set of morphological characters common to all species of the genus *Portunus* that allow distinguishing its megalopas from those of other genera of portunids. However, there are some characters that can be used to distinguish the megalopas already described, such as: presence of carpal spine in *P. hastatus*, *P. pelagicus* and *P. gibbesii* and absence in *P. trituberculatus*; ischial hook on cheliped present in *P. pelagicus* and *P. trituberculatus,* but absence in *P. hastatus* and *P. gibbesi*; and the 8-segmentation of the antennal segment in *P. hastatus* and *P. pelagicus* while it is 7-segmented in *P. gibbesii.*

*Charybdis (Charybdis) hellerii* (Figs. 2d, 4d, j, 8d).

There are published megalopas descriptions for seven species of *Charybdis*: *C. japonica* (A. Milne Edwards, 1861) by Kurata and Nishina^53^, *C. acuta* (A. Milne Edwards, 1869) by Kurata and Omi^54^, *C. callianassa* (Herbst, 1789) by Greenwood and Fielder^47^, *C. truncata* (Fabricius, 1798) by Greenwood and Fielder^55^, C. *feriata* (Linnaeus, 1758) by Fielder et al.^56^, *C. bimaculata* (Miers, 1886) by Hwang and Kim^57^ and *C. natator* (Herbst, 1794) by Islma et al.^58^.

Dineen et al.^59^ described the larval development of *C. hellerii* but no provided detailed morphological description of the megalopa stage, only one photograph. Kurata and Nishina^52^ described the megalopas of *C. acuta* and *C. japonica,* collected in Japan, but they are too brief to allow detailed comparison with other species of *Charybdis* (see Table 4).

**Table 4:** Useful meristic features to compare the megalopa stage of the genera *Charybdis*, *Thalamita* and *Callinectes.*

	Antennal flagellum (s)	Endopod of maxillule (s)	Uropod (s)	Ischial spine on cheliped
*Charybdis callianassa*^47^	4,0,2,1,3,0,3,4	7	–	Present
*Charybdis feriata*^56^	0,0,4,3,4,2,4,5	5	1,12	Absent
*Charybdis truncata*^55^	0,0,4,2,2,2,5,5	5	1,11	Present
*Charybdis bimaculata*^57^	0,0,4,2,5,1,3,4	6	1,8	Present
*Charybdis natator*^58^	0,0,2,2,5,2,5,4	6	1,11	Present
*Thalamita danae*^60^	0,0,4,1,5,1,3,5	5	–	Absent
*Thalamita crenata*^61^	0,2,0,0,3,1,2,4	5	1,11	Absent
*Thalamita pelsarti*^62^	0,0,3,2,4,2,2,3	6	1,11	Absent
*Callinectes sapidus*^63^	0,0,4,2,5,1,3,3	4,4	1,11	Present
*Callinectes similis*^64^	0,0,4,2,5,3,4,4	5	0,11	Present

*s* setation.

Although, the earlier published larval descriptions lack enough detail, it is noted that these species share the same external characters, such as: antennal flagellum 8-segmented, absent of carpal spine and ischial hook on cheliped (absent in *C. feriata*), and a coxal spine on 2^nd^ pereiopod (absent in *C. feriata*).

*Thalamita admete* (Figs. 2e, 4e, k, 8e)

In the genus *Thalamita*, descriptions of the megalopas are available for *T. danae* Stimpson, 1858 by Fielder and Greenwood^60^, *T. crenata* Rüppell, 1830 by Krishnan and Kannupandi^61^, and *T. pelsarti* Montgomery, 1931 by Islam et al.^62^.

We compared them and found that all species share the absence of spines on cheliped and pereiopods. These characters combined with an 8-segmented antennal flagellum and setation of uropods (1,11) characterize the genus. *Thalamita admete* can be differentiated from the three species by antennal setation (Table 4).

*Callinectes* spp. (Figs. 2f–I, 4f, l–o, q, r, 8f–i)

The megalopa stage known of *Callinectes* are: *C. sapidus* Rathbun, 1896 by Costlow and Bookhout^63^, and *C. similis* Williams, 1966 by Bookhout and Costlow^64^. The authors differenced the megalopa stage of these two species by size and examination of minute characteristics, but larval descriptions lack enough detail.

In this study megalopas of four species of the *Callinectes* are described*: C. amnicola, C. arcuatus, C. ornatus*, and *C. toxotes* as shown in Fig. 2f–i. The megalopas of *Callinectes* genus are strongly similar, being difficult to differentiate them only based on external characters. These six species share the most important external characters like the antennal flagellum 8-segmented, carpal spine on cheliped absent, ischial hook on cheliped present and coxal spine on 2^nd^ pereiopod absent. Even the carapace with tubercle on protogastric region with a row of 8–10 minute setae is the same for the four *Callinectes* species of this study.

The four species can be differentiated only by a thorough examination of the mouthparts (see Tables 2 and 4).

Family Xanthidae MacLeay, 1838

We have identified the megalopa stage of 5 species of xanthids. Xanthidae is a large and heterogeneous family containing about 124 genera and around 640 species^47,58^. The intergeneric variation in xanthids megalopas appears to be too significant to find constants group characters, as in adults^40,65^.

In this paper is presented for the first time morphological features of the megalopa stage for the genera *Neoliomera* Odhner, 1925*, Liomera* Dana, 1851 (Figs. 5b, 7b, g, 8k), *Pseudoliomera* Odhner, 1925 (Figs. 5c, 7c, h, o, n, 8l), and *Williamstimpsonia* Števčić, 2011 (Figs. 5d, 7d, i, l, m, 8m) (Table 3). The lack of previous descriptions of the megalopas of other species belonging to these genera does not allow a review of morphological characteristics.

*Etisus odhneri* (Figs. 5f, 7f, k, m, 8o)

In this genus, the only larval development known is for *Etisus laevimanus* Randall, 1840 by Suzuki^66^. We compared them and found several minute differences, all related with the number of setae in antennule, antenna, and uropods. Antennule accessory flagellum in *E. odhneri* presents 1 + 1 + 3 setae and in *E. laevimanus* 1 + 3 setae; antennal flagellum setation in *E. odhneri* is 0,0,3,0,3,0,4,4 and 0,0,2–3,0,4,0,4,4 in *E. laevimanus;* and uropod in *E. odhneri* presents 0,10 setae and 1, 10–11 in *E. laevimanus*. Both species show ischial spine on cheliped.

Family Pseudorhombilidae Alcock, 1900

Pseudorhombilidae includes 19 genera and 50 species^67^, but larval data are only known for 3 species. In the present study only one megalopa of the monospecific genus *Scopolius* have been identified, *S. nuttingi* (Figs. 5e, 7e, j, 8n), that lack of previous larval descriptions.

## Discussion

DNA barcoding is a useful tool for the identification of crustaceans by assigning indeterminate specimens to known species^11,68,69^ and faster method for the descriptions of brachyuran larvae^13^. As more research uses these genetic markers begin to be addressed questions relating to the biodiversity, ecology, and evolution of natural systems^70,71^.

But, although this molecular technique is widely used and popular, identifying unknown specimens through DNA barcodes requires a reference library containing morphologically-identified barcoded specimens against which unknowns can be compared^72^, highlighting the reliability of the database with adequate validation and detection of erroneous sequences^73,74^. While the use of DNA barcode databases for the identification of many marine species is an increasingly used technique in taxonomy, there are still large numbers of unexplored taxa, with little or no DNA barcode coverage or, sometimes, several species lack of sequence in the database is correctly assigned to higher taxa^75,76^. Thus, the results provide useful information to estimate species numbers, regardless of their formal taxonomic state, distribution and ecology as well as a framework for future taxonomic work^77,78^.

The availability of this information, especially for the family Portunidae, is of great importance not only to understand the life history of these species that are of commercial and therefore economic interest, but also because they have invasive potential. Species belonging to this family has a high dispersal potential because the adults are swimmers, have long larval periods and all stages of the life cycle can actively migrate long distances and, therefore, are dispersal agents both within the region of origin and to new environments^79^, which can turn them into invasive species, as is the case of the blue crab, *Callinectes sapidus,* in the Mediterranean.

In the present work, *Charybdis hellerii* is the only one portunid that has been reported as an invasive crab^80^ where it was collected, and this species continues to expand its range^81^ in Caribbean Sea. However, although all the other Portunidae megalopas were collected within their known range, it is interesting to note that they were collected several miles from the coast, in the open sea, which highlights the potential to expand their range of distribution.This work focused mainly on the taxonomic applications of DNA barcoding to increase the knowledge of unknown brachyuran megalopa stages. This study provides a valuable larval morphology information about 9 portunids, 5 xanthids and 1 pseudorhombilid that will support future systematic, ecological, and biological studies about these families.

## Methods

### Fieldwork

Megalopas were collected in two research cruises under the support of the MALASPINA 2010–2011 and MAF 2015 research projects (Table 5). The MALASPINA Circumnavigation Expedition was carried out with the general objectives of assessing the impact of global change on the oceans and exploring its biodiversity. The research cruise was conducted between December 2010 and July 2011, involved two oceanographic research vessels, the *Hespérides* and the *Sarmiento de Gamboa,* which covering a total of 42,000 nautical miles through the tropical and subtropical regions of the Atlantic, Indian, and Pacific oceans, sampling in a total of 147 stations. MAF research cruise was held to assess the carbon vertical active flux in the open sea due to zooplankton and micronekton and main responsible species. A total of 13 stations were sampled between 3^rd^ and 29^th^ April 2015 on board of the RV *Hespérides*, which crossed the tropical and subtropical Atlantic regions from Salvador de Bahia, Brazil, to Las Palmas, Canary Islands, Spain. (Fig. 1).

**Table 5:** Sampling sites (geographical coordinates), project, and number of the megalopas of the species of portunids, pseudorhombilid and xanthids collected in the MALASPINA 2010–2011 and MAF 2015 expeditions.

Species	N	Latitude	Longitude	Project
**Family Portunidae**				
*Achelous floridanus*	5	03° 10′ 48" S	28° 26′ 39" W	MAF
*Arenaeus mexicanus*	1	07° 13′ 25" N	87° 57′ 35" W	MALASPINA
*Callinectes amnicola*	4	10° 52′ 02" N	22° 38′ 36" W	MAF
*Callinectes arcuatus*	2	10° 05′ 33" N	99° 14′ 46" W	MALASPINA
*Callinectes arcuatus*	1	07° 13′ 25" N	87° 57′ 35" W	MALASPINA
*Callinectes arcuatus*	1	09° 26′ 44" N	96° 20′ 26" W	MALASPINA
*Callinectes arcuatus*	1	08° 08′ 31" N	90° 21′ 54" W	MALASPINA
*Callinectes ornatus*	1	22° 57′ 17" S	36° 55′ 29" W	MALASPINA
*Callinectes toxotes*	1	07° 13′ 25" N	87°57′35" W	MALASPINA
*Charybdis (Charybdis) hellerii*	2	15°04′07" N	69° 17′ 43" W	MALASPINA
*Portunus (Portunus) hastatus*	5	16° 09′ 36" N	26° 01′ 48" W	MALASPINA
*Thalamita admete*	1	34° 26′ 23" S	31° 06′ 43" E	MALASPINA
**Family Pseudorhombilidae**				
*Scopolius nuttingi*	1	17° 25′ 38" N	59° 49′ 40" W	MALASPINA
**Family Xanthidae**				
*Etisus odhneri*	1	34° 50′ 14" S	27° 32′ 57" E	MALASPINA
*Liomera cinctimanus*	1	34° 10′ 26" S	33° 43′ 33" E	MALASPINA
*Neoliomera cerasinus*	1	35° 08′ 10" S	25° 33′ 47" E	MALASPINA
*Pseudoliomera variolosa*	1	20° 20′ 40" N	145° 11′ 50" W	MALASPINA
*Williamstimpsonia stimpsoni*	1	07° 13′ 25" N	87° 57′ 35" W	MALASPINA

### Sample processing

In both research expeditions, megalopas were collected from the superficial layer with a neuston net with a mesh size of 200 microns, hauled from 10 to 15 min at 2–3 knots. Samples were immediately fixed and preserved in 95% ethanol. All brachyuran megalopa stages were counted and sorted from the zooplankton samples using a stereomicroscope. Prior to DNA extraction, all larvae were examined morphologically and sorted into morphotypes according to the external characters.

### Megalopas morphological descriptions

For easier observation of larvae structures and setation under microscope, megalopas were first placed for 5–10 min in a watch glass with 2 ml of warm lactic acid before proceeding with the dissection of the body parts^82^.

Drawings and measurements of megalopa stage were made using *a Leica MZ6* and microscope *Nikkon Eclipse 90i* with integrated *camera lucida*. All measurements were made using an ocular micrometer. Descriptions were based on all the collected megalopas of each species identified by DNA barcoding (see Table 1). The following measurements were taken for the megalopa: cephalothorax length (CL), measured from the rostrum (tip of rostrum in portunids) to posterior margin of cephalothorax; and cephalothorax width (CW), measured as the cephalothorax maximum width (mesobranchial regions).

For the megalopas dorsal view, only the left pereiopods were drawn since one of the right pereiopods was used for molecular analyzes.

Descriptions were arranged according to the standards proposed by Clark et al.^83^ and Clark & Cuesta^84^, and setal terminology follows the classification by Landeira et al.^85^. A detailed description of *Achelous floridanus* is provided while the others portunids descriptions are summarized in the Table 2. For Xanthidae and Pseudorhombilidae families, the specie of *Neoliomera cerasinus* is described in detail and the others xanthoids descriptions are summarized in the Table 3.

### Molecular analysis

The identification of the megalopas was based on partial sequences of the 16S rRNA and COI mitochondrial genes. Total genomic DNA of the megalopas from MALASPINA Expedition was extracted from muscle tissue from one pereiopod and incubated for 1–24 h in 300 µl lysis buffer (5 ml of 1 M Tris–HCl (pH 8), 1 ml 0.5 M EDTA, and 5 ml of 10% SDS solution to 400 ml of distilled water) at 65º C. Protein was precipitated by addition of 100 µl of 7.5 M ammonium acetate and subsequent centrifugation, and DNA precipitation was obtained by addition of 300 µl of isopropanol and posterior centrifugation. The resulting pellet was washed with ethanol (70%), dried, and finally resuspended in Milli-Q distilled water^82^. In the megalopas from MAF Expedition, total genomic DNA was also extracted from muscle tissue from one pereiopod, but the extraction process followed a modified Chelex 10% protocol by Estoup et al.^86^. Target mitochondrial DNA from the 16S rRNA and COI genes was amplified with the primers and the cycling conditions of the polymerase chain reaction (PCR) listed in Table 6. PCR products were sent to New Biotechnic, CISA-INIA, and Stab Vida companies to be purified and then bidirectionally sequenced.

**Table 6:** Data of sequenced genes, 16S and COI, including primers used for each gene, and reference (Ref), cycling conditions of the PCR, in all cases: initially 2 min at 95 °C and finally 5 min at 72 °C (35–40 cycles), and length, number of base pairs, of the sequences obtained (bp).

Genes	Primers	Ref.	Pair	PCR cycling conditions	bp
16S	16S 1472: (5′-AGATAG AAA CCA ACC TGG-3′)	^87^	16S L2	20 s – 95 °C, 20 s – 45–48 °C, 45 s − 72 °C	540
	16S L2: (5′-TGC CTG TTTATC AAA AAC AT-3′)	^88^			
COI	COH6 (5´- TAD ACT TCD GGR TGD CCA AAR AAY CA -3´)	^89^	COL6b	20 s – 95 °C, 20 s – 45–48 °C, 47 s − 72 °C	670
	COL6b (5′-ACA AAT CATAAA GATATY GG-3′)	^89^			

Sequences were edited using the software Chromas version 2.0. With the obtained final DNA sequences were performed a BLAST (Basic Local Alignment Search Tool) on NCBI (National Center for Biotechnology Information) web facility on GenBank sequences database (http://www.ncbi.nlm.nih.gov/genbank/) to get the best matches for identification. The COI sequences were also searched in the official Barcode of Life database (BOLD) (http://v3.boldsystems.org/index.php/IDS_OpenIdEngine). Identifications were considered as positive when retrieved sequences showed similarity values greater than 99%, only differed in 1–3 or 1–7 mutations in 16S or COI, respectively, a more conservative limit than other previous works identifying decapod larvae considering > 98%^96^. Larval sequences for both genes are deposited in Genbank (see Table 1).

## Ethical approval

This article does not contain any studies with human participants performed by any of the authors. All applicable international, national, and institutional guidelines for the care and use of animals were followed.
